# Development and validation of a nomogram for breast cancer-related lymphedema

**DOI:** 10.1038/s41598-024-66573-1

**Published:** 2024-07-06

**Authors:** Qihua Jiang, Hai Hu, Jing Liao, Zhi-hua Li, Juntao Tan

**Affiliations:** 1 Department of Breast Surgery, Third Hospital of Nanchang, No. 2, Xiangshan South Road, Xi Hu District, Nanchang City, 330008 Jiangxi Province China; 2 Department of General Surgery, Third Hospital of Nanchang, No. 2, Xiangshan South Road, Xi Hu District, Nanchang City, 330008 Jiangxi Province China; 3 Jiangxi Province Key Laboratory of Breast Diseases, Third Hospital of Nanchang, No. 2, Xiangshan South Road, Xihu District, Nanchang City, 330008 Jiangxi Province China

**Keywords:** Breast cancer-related lymphedema, Risk factors, Nomogram, Predictive model, Medical research, Oncology

## Abstract

To establish and validate a predictive model for breast cancer-related lymphedema (BCRL) among Chinese patients to facilitate individualized risk assessment. We retrospectively analyzed data from breast cancer patients treated at a major single-center breast hospital in China. From 2020 to 2022, we identified risk factors for BCRL through logistic regression and developed and validated a nomogram using R software (version 4.1.2). Model validation was achieved through the application of receiver operating characteristic curve (ROC), a calibration plot, and decision curve analysis (DCA), with further evaluated by internal validation. Among 1485 patients analyzed, 360 developed lymphedema (24.2%). The nomogram incorporated body mass index, operative time, lymph node count, axillary dissection level, surgical site infection, and radiotherapy as predictors. The AUCs for training (N = 1038) and validation (N = 447) cohorts were 0.779 and 0.724, respectively, indicating good discriminative ability. Calibration and decision curve analysis confirmed the model’s clinical utility. Our nomogram provides an accurate tool for predicting BCRL risk, with potential to enhance personalized management in breast cancer survivors. Further prospective validation across multiple centers is warranted.

## Introduction

Breast cancer remains one of the most prevalent malignancies among women worldwide, with treatment modalities encompassing surgery, chemotherapy, radiotherapy, and endocrine therapy^1–3^. Thanks to early detection, improved treatments, and multidisciplinary rehabilitation approaches, breast cancer patients now experience a five-year survival rate of 89.7%^4^. Patients recovering from breast cancer surgery often hold substantial expectations for their postoperative quality of life; nevertheless, they are frequently confronted with the prevalent complication of breast cancer-associated lymphedema (BCRL). This condition manifests as localized swelling, predominantly affecting the unilateral or bilateral upper limbs, attributable to the abnormal retention of lymphatic fluid with a high protein content within the surrounding soft tissues^5^. The occurrence of BCRL can be seen in 20 to 75% of patients, mostly within two years after breast cancer treatment, but it can arise later as well^6^. BCRL can cause physical issues like swelling and pain, and it can significantly impede daily activities. It can also lead to emotional distress, including anxiety and depression, greatly affecting the patients’ well-being^7–9^.

Previous research efforts have focused on pinpointing various risk factors associated with BCRL, including surgical procedures with an emphasis on axillary lymph node dissection (ALND), the application of radiotherapy and chemotherapy, along with patient-specific factors like tumor dimensions, age, and body mass index (BMI)^10,11^. While these studies have provided crucial insights into understanding lymphedema, there remains an absence of comprehensive assessment tools that integrate multiple factors to predict an individual patient’s risk of lymphedema.

To address this gap, our study aims to establish and validate a novel, multifactorial risk prediction model for BCRL, incorporating not only established risk factors but also operative time, a factor not thoroughly examined in previous models. A nomogram, a graphical risk prediction tool that combines multiple prognostic factors to offer personalized risk assessment for patients, will be utilized in this study^12^. By assigning numerical scores to pertinent factors and summing them, a total score can be calculated to predict the likelihood of a specific medical outcome^13^. Prior risk prediction models for BCRL have relied on factors related to patient health and treatment regimens, yet they have not undergone comprehensive validation^14–16^. Furthermore, few models have considered operative time, a factor closely associated with BCRL^17^. Studies have indicated that longer operative times may increase the risk of lymphedema due to extended tissue manipulation and potential for greater lymphatic disruption^17,18^.

The primary objective of this study is to establish the multifactorial risk factors for BCRL by analyzing demographic data, disease and treatment characteristics, as well as operative time, and to develop and validate a nomogram for predicting the risk of BCRL. We hypothesize that operative time could act as an independent risk factor for BCRL, suitable for use in developing a predictive nomogram. By focusing on the integration of operative time with other established risk factors, our study aims to provide a more comprehensive and validated tool for predicting BCRL, thereby offering new insights and practical applications for improving patient outcomes in breast cancer care.

## Materials and methods

### Study population

**Figure 1:**
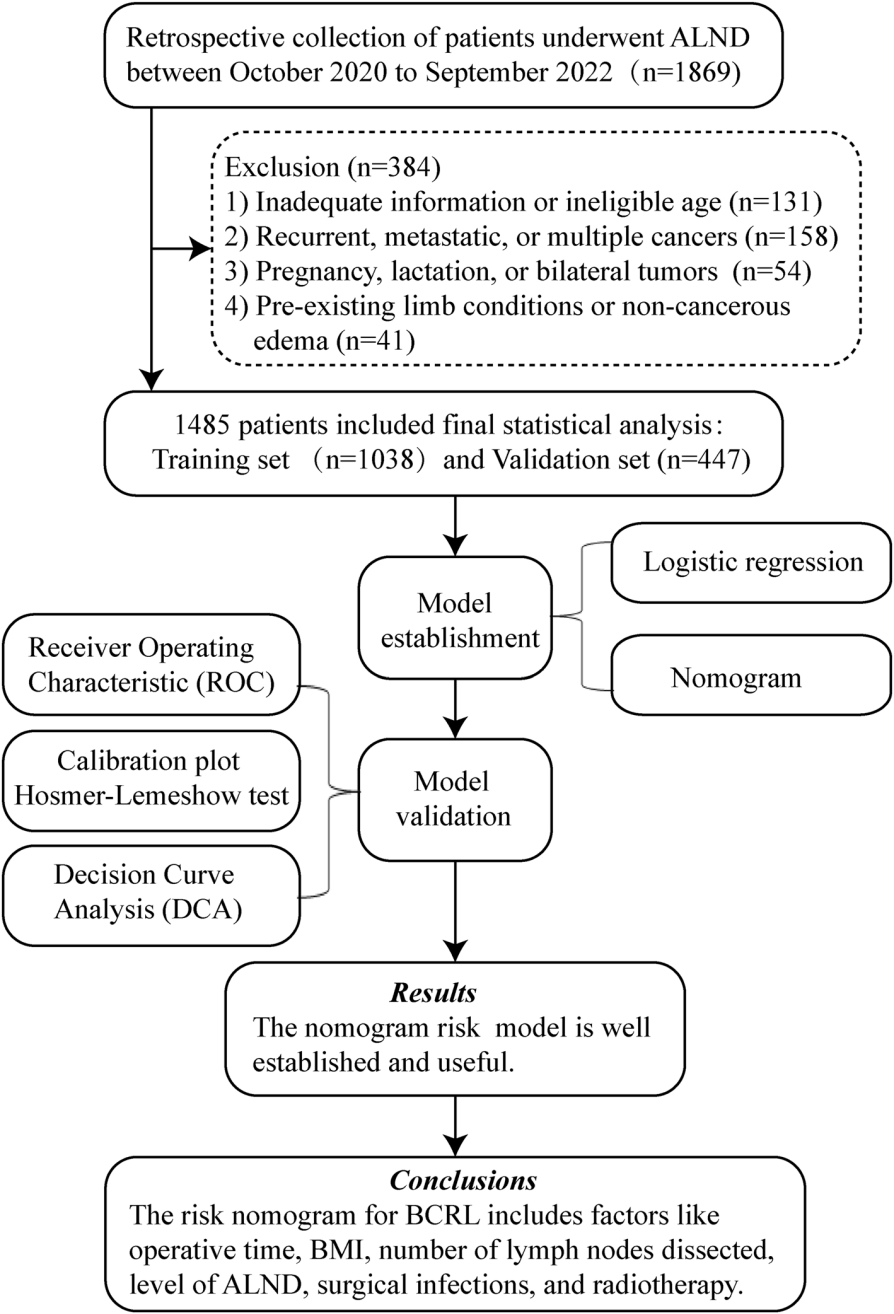
The workflow of this study. *BMI* body mass index, *ALND* axillary lymph node dissection, *BCRL* breast cancer-related lymphedema.

From October 2020 to September 2022, breast cancer patients treated with ALND (level I, II, or III) were included from the patient population at the Third Hospital of Nanchang. We included adults with proven breast cancer who had surgery and complete medical records. Exclusions were for those with returning or spread cancer, other tumors, pregnancy, bilateral breast cancer, serious other illnesses, prior surgeries on the same limb, or non-cancer related swelling. Figure 1 illustrates the workflow of this study. Ethical approval was obtained from the hospital’s ethics committee, adhering to the Helsinki Declaration.

### Data selection

Data was extracted from the patients’ electronic medical records, including demographic characteristics (such as age, BMI at surgery, history of hypertension and diabetes), disease features (such as clinical staging), treatment information (such as type of surgery, radiotherapy, chemotherapy, endocrine therapy), operative time, number of lymph nodes dissected, level of ALND, surgical site infection, and the occurrence of postoperative lymphedema. Surgical site infection after breast cancer surgery is defined as infections occurring at axillary surgical site within 30 days post-operation, characterized by symptoms like swelling, redness, and discharge^19^.

For the radiotherapy group, we collected detailed information regarding the type and extent of radiotherapy administered to the study participants. Specifically, we recorded whether patients underwent axillary field radiotherapy, which targets the lymph nodes in the axilla region. Additionally, we categorized radiotherapy as either targeting the breast only, axillary only, or both breast and axillary regions (Supplementary Table 1).

Data regarding chemotherapy were collected from the electronic medical records of the patients. This included information on the type of chemotherapy administered (e.g., anthracyclines, taxanes, cyclophosphamide, etc.), as well as the timing of chemotherapy in relation to surgery (neoadjuvant, adjuvant, or both). The specific regimens, doses, and duration of chemotherapy were documented to understand their potential impact on the development of BCRL (Supplementary Table 1).

Operative time was defined as the total duration from the first incision to the final suture for each individual surgery. If a patient underwent more than one operation, the operative times were recorded separately for each surgery and not summed. To classify patients into operative times of less than or more than 2 h, the longest operative time from multiple surgeries was used.

### Measurement of lymphedema

To establish the presence of lymphedema, circumferences were measured at four anatomical landmarks: the metacarpal-phalangeal joints, the wrists, and locations 10 cm distal and 15 cm proximal to the lateral epicondyle. Lymphedema was diagnosed when a measurement exceeded that of the opposite limb by 2 cm or more at any of these reference points^20^.

Patients underwent regular lymphoedema screening post-surgery, with measurement time points at 1, 3, 6 and 12 months postoperatively, followed by every 6 months until the end of the study. These regular measurements allowed for accurate assessment of the onset and progression of lymphedema^6^.

### Statistical analyses

Statistical evaluations were conducted using [R software (version 4.1.2)] (https://cran.r-project.org/bin/windows/base/old/4.1.2/) and [IBM SPSS Statistics (version 25.0)](https://www.ibm.com/support/pages/downloading-ibm-spss-statistics-25). The study sample of 1485 breast cancer patients were randomly segmented into a 7:3 training-to-validation set. Average values and standard deviations represented continuous data, while percentages described categorical data. We applied logistic regression analyses to uncover factors influencing lymphedema after surgery. Only variables with p-values under 0.05 moved to multivariate analysis for independent predictor identification. We then constructed a nomogram, assessed its accuracy with the receiver operating characteristics (ROC) curves, and validated it with calibration techniques and decision curve analysis (DCA). We considered p-values below 0.05 to denote statistical significance.

### Ethics statement

The subjects included in this study were human participants, and the research was approved by the Ethics Committee of the Third Hospital of Nanchang City, also known as the Institutional Review Board (IRB) of the Third Hospital of Nanchang. Patients provided written informed consent to participate in this study.

## Results

### Patient characteristics

This retrospective study, adhering closely to predefined criteria, culminated in a total pool of 1485 patients for the definitive analysis. Of these, 360 were diagnosed with lymphoedema and 1125 without lymphoedema. The division of patients into two distinct groups was executed at random, resulting in 1038 individuals in the training set and 447 in the validation set. Table 1 presents a summary of their baseline characteristics.

**Table 1:** Patient characteristics for the 1485 study participants, stratified by lymphoedema and divided into training and validation sets.

Characteristic	Total (n = 1485), N (%)	Training set (n = 1038), N (%)	Validation set (n = 447), N (%)	Lymphoedema (n = 360), N (%)	Non-lymphoedema (n = 1125), N (%)	P Le vs. non-le
Age (years) < 60 ≥ 60	1308 (88.1) 177 (11.9)	915 (88.2) 123 (11.8)	393(87.9) 54 (12.1)	309 (85.8) 51 (14.2)	999 (88.8) 126 (11.2)	0.131
BMI (kg/m^2^) < 25 ≥ 25	1080 (72.7) 405 (27.3)	708 (68.2) 330 (31.8)	372 (83.2) 75 (16.8)	213 (64.2) 129 (35.8)	849 (75.5) 276 (24.5)	< 0.001
Hypertension Yes No	261 (17.6) 1224 (82.4)	207 (19.9) 831 (80.1)	54 (12.1) 393 (87.9)	69 (19.2) 291 (80.8)	192 (17.1) 933 (82.9)	0.362
Diabetes Yes No	135 (9.1) 1350 (9.1)	108 (10.4) 930 (89.6)	27 (6.0) 420 (94.0)	39 (10.8%) 321 (89.2%)	118 (10.5%) 1007 (89.5%)	0.853
Staging (UICC) 0 I II III	24 (1.6) 168 (11.3) 1035 (69.7) 258 (17.4)	18 (1.7) 132 (12.7) 780 (75.1) 108 (10.4)	6 (1.3) 36 (8.1) 255 (57.0) 150 (33.6)	6 (1.7) 30 (8.3) 240 (66.7) 84 (23.3)	18 (1.6) 146 (13.0) 738 (65.6) 223 (19.8)	0.084
Type of surgery Conservative Mastectomy Breast reconstruction	351 (23.6) 1104 (74.4) 30 (2.0)	243 (23.4) 777 (74.9) 18 (1.7)	114 (25.5) 321 (71.8) 12 (2.7)	81 (22.5) 270 (75.00) 9 (2.5)	276 (24.5) 828 (73.6) 21 (1.9)	0.582
Surgery dominant arm Yes No	840 (56.6) 645 (43.4)	603 (58.1) 435 (41.9)	237 (53.0) 210 (47.0)	198 (55.0) 162 (45.0)	642 (57.1) 483 (42.9)	0.491
Operation time (hours) < 2 ≥ 2	1302 (43.4) 183 (12.3)	933 (89.9) 105 (10.1)	369 (82.6) 78 (17.4)	285 (79.2) 75 (20.8)	1017 (90.4) 108 (9.6)	< 0.001
Number of lymph nodes dissected < 10 10–20 > 20	369 (24.8) 960 (64.6) 156 (10.5)	234 (22.5) 675 (65.0) 129 (12.4)	135 (30.2) 285 (63.8) 27 (6.0)	51 (14.2) 243 (67.5) 66 (18.3)	318 (28.3) 717 (63.7) 90 (8.0)	< 0.001
Level of ALND I II III	99 (6.7) 741 (49.9) 645 (43.4)	72 (6.9) 528 (50.9) 438 (42.2)	27 (6.0) 213 (47.7) 207 (46.3)	3 (0.8) 120 (33.3) 237 (65.8)	96 (8.5) 621 (55.2) 408 (36.3)	< 0.001
Surgical site infection Yes No	330 (22.2) 1155 (77.8)	240 (23.1) 798 (76.9)	90 (20.1) 357 (79.9)	123 (34.1) 237 (65.9)	207 (18.4) 918 (81.6)	< 0.001
Chemotherapy Yes No	1410 (95.0) 75 (5.0)	972 (93.6) 66 (6.4)	438 (98.0) 9 (2.0)	345 (95.8) 15 (4.2)	1065 (94.6) 60 (5.4)	0.379
Radiotherapy Yes No	393 (26.5) 1092 (73.5)	261 (25.1) 777 (74.9)	132 (29.5) 315 (70.5)	147 (40.8) 213 (59.2)	246 (21.9) 879 (78.1)	< 0.001
Hormonal therapy Yes No	486 (32.7) 999 (67.3)	312 (30.1) 726 (69.9)	174 (38.9) 273 (61.1)	129 (35.8) 231 (64.2)	357 (31.7) 768 (69.3)	0.149

*Le* Lymphoedema, *non-le* non-lymphoedema, *BMI* body mass index, *ALND* axillary lymph node dissection.

Age was similar across groups, with no significant age difference (P = 0.131). There was a statistically significant higher occurrence of elevated BMI (≥ 25 kg/m^2^) among patients with lymphoedema, at a rate of 35.8%, versus only 24.5% in those without lymphoedema (P < 0.001). Comorbidities (hypertension or diabetes) and cancer staging showed no significant difference between groups. Type of surgery and surgery on the side of the dominant arm were not significant factors. Longer operative time (over 2 h) was linked to a higher risk of lymphoedema (20.8% vs. 9.6%, P < 0.001), as was a greater number of lymph nodes dissected (P < 0.001). Higher levels of ALND and surgical site infection were significantly associated with lymphoedema (P < 0.001). Of the adjuvant therapies, only radiotherapy showed a higher prevalence in the lymphoedema group (40.8% vs. 21.9%, P < 0.001). These findings indicate that longer surgical duration, higher BMI, more lymph nodes dissected, higher levels of ALND, surgical site infection, and radiotherapy are risk factors for developing lymphoedema post-breast cancer surgery.

### Logistic regression for BCRL

Significant predictors from univariate analysis were entered into a subsequent multivariate logistic regression model. The findings revealed that BMI, operative time, number of lymph nodes dissected, level of ALND, surgical site infection, and radiotherapy were independent risk factors for BCRL, as detailed in Table 2.

**Table 2:** Logistic regression analysis of the predictors for the risk of BCRL.

Variables	OR (95%CI)	P
BMI (≥ 25 vs. < 25 kg/m^2^)	1.73 (1.30–2.31)	< 0.001
Operation time (≥ 2 vs. < 2 h)	2.85 (1.98–4.12)	< 0.001
Number of lymph nodes dissected		
10–20 vs. < 10	2.57 (1.79–3.67)	< 0.001
> 20 vs. < 10	5.65 (3.50–9.13)	< 0.001
Level of ALND		
II vs. I	4.86 (3.33–6.60)	< 0.001
III vs. I	7.89 (2.81–16.33)	< 0.001
Surgical site infection (Yes vs. No)	2.20 (1.64–2.96)	< 0.001
Radiotherapy (Yes vs. No)	2.28 (1.72–3.03)	< 0.001

*BCRL* breast cancer-related lymphedema, *BMI* body mass index, *ALND* axillary lymph node dissection.

### Development of the prediction model for BCRL

**Figure 2:**
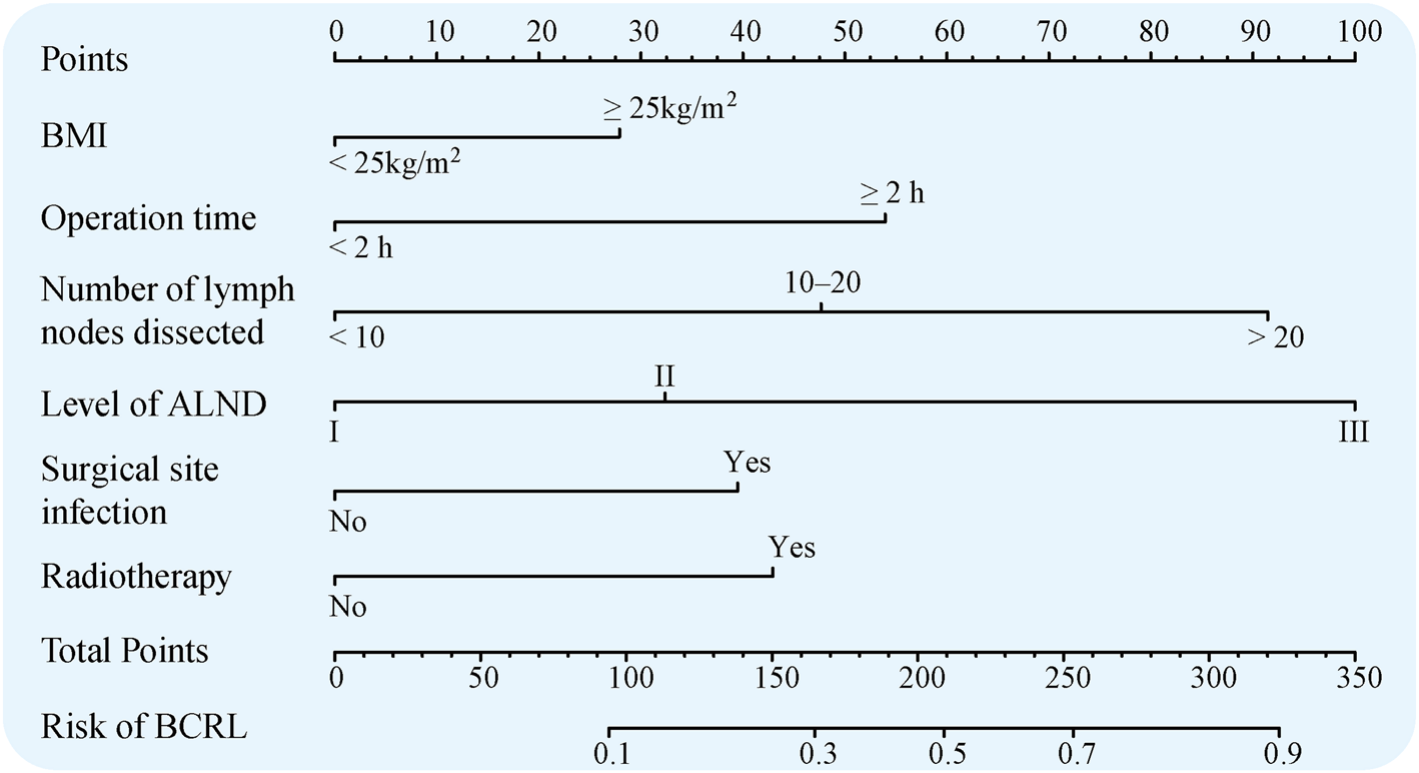
The graph showed nomogram for predicting risk of BCRL. *BMI* body mass index, *ALND* axillary lymph node dissection, BCRL breast cancer-related lymphedema.

The final predictive model for BCRL incorporated six variables identified as independent prognostic factors. These variables served as predictors, with individualized nomograms developed using R software (version 4.1.2). The contribution of each predictor was quantified based on regression coefficients obtained from the final model analysis (Fig. 2). The model allocated scores as follows: BMI < 25 kg/m^2^ (0 points), BMI ≥ 25 kg/m^2^ (27.5 points); operative time < 2 h (0 points), ≥ 2 h (53.75 points); number of lymph nodes dissected: < 10 (0 points), 10–20 (47.5 points), > 20 (92.5 points); Level of ALND: I (0 points), II (32.5 points), III (100 points); surgical site infection: no (0 points), yes (40 points); radiotherapy: no (0 points), yes (43.75 points). The total score, representing the sum of the points from these six items, correlates with the individual risk of developing BCRL.

### Validation of the prediction model

**Figure 3:**
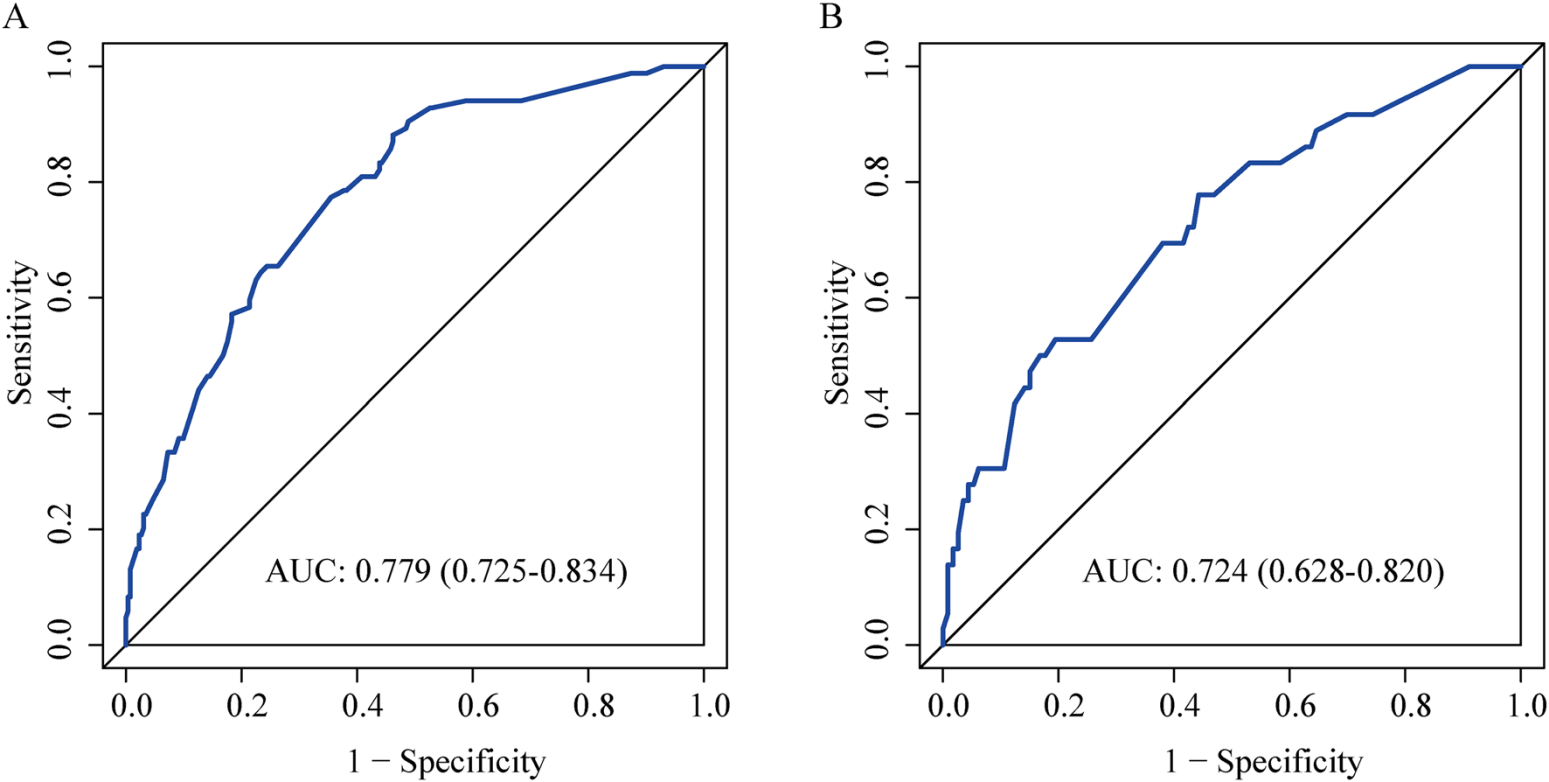
Validation of ROC curves for the nomogram model to predict BCRL risk in the training set (**A**) and validation set (**B**).

**Figure 4:**
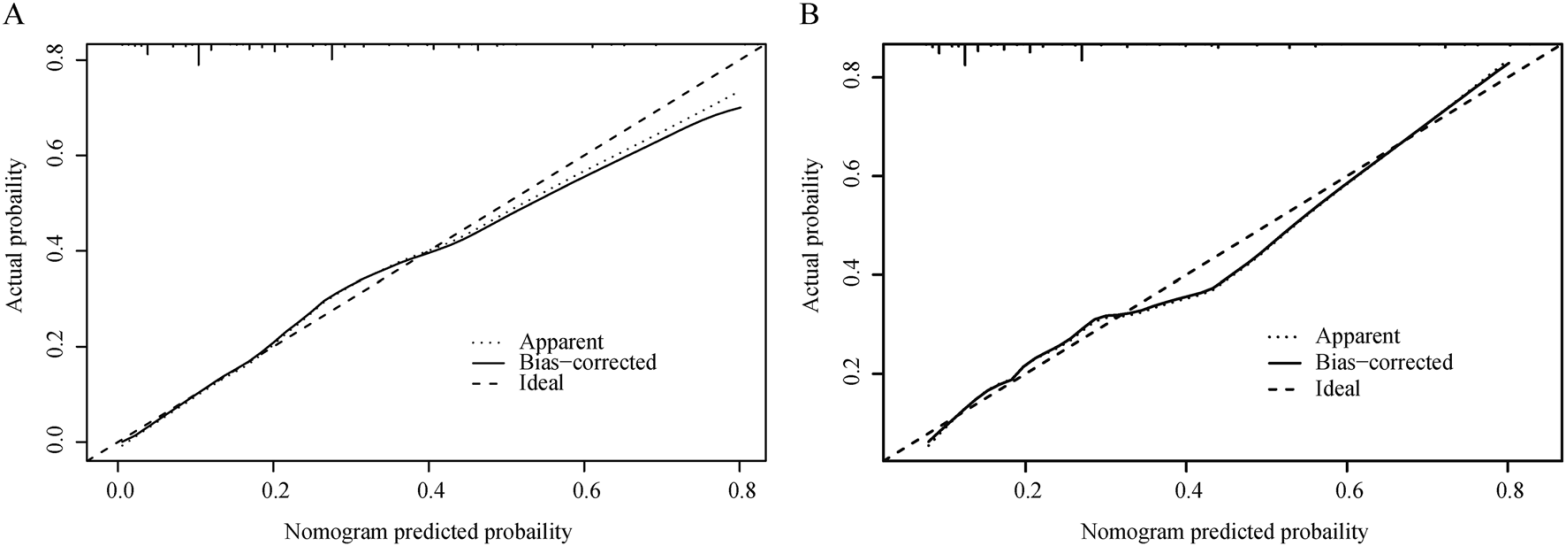
Calibration of the nomogram model for predicting BCRL risk in the training set (**A**) and validation set (**B**).

**Figure 5:**
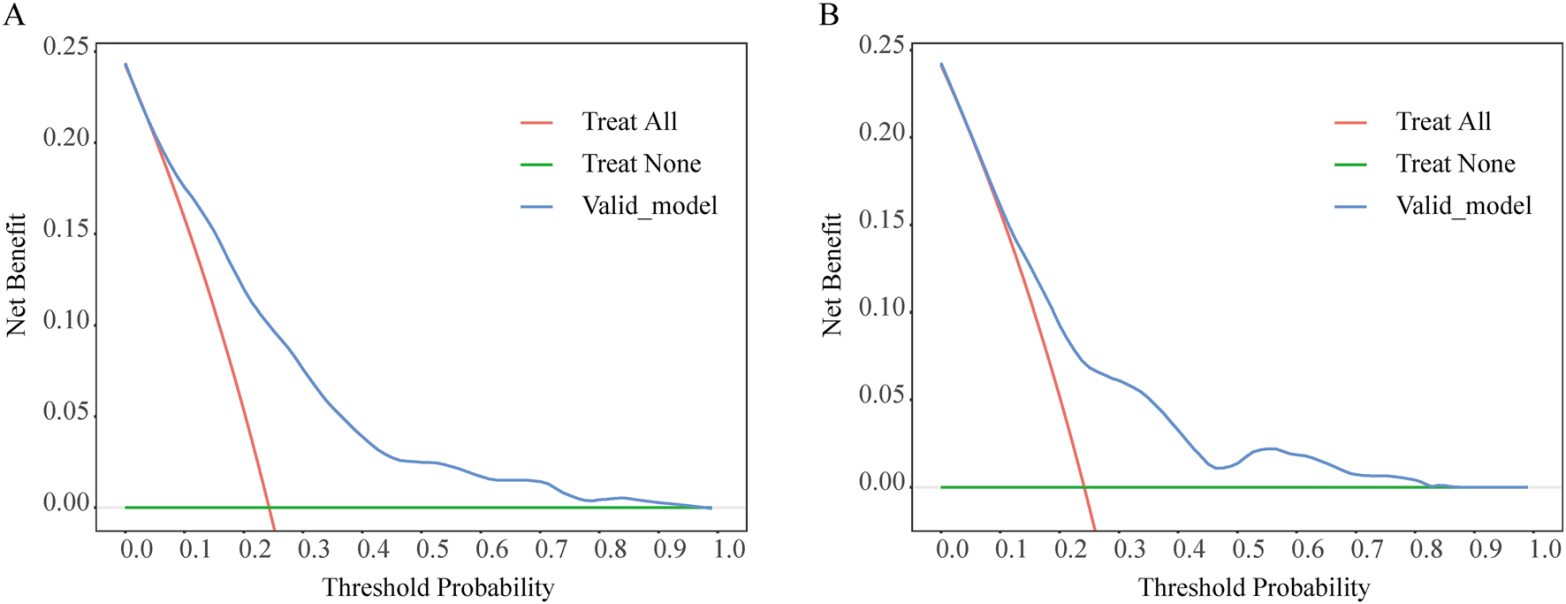
Decision analysis curves for the nomogram model for predicting BCRL risk in the training set (**A**) and validation set (**B**).

The ROC curve analysis was utilized to measure the predictive model’s differentiation capability, with AUC results of 0.779 for the training set and 0.724 for the validation set, denoting moderately high predictiveness (Fig. 3). The predictive model was verified with calibration curve and the Hosmer-Lemeshow test. The calibration plots suggested a very good fit of the predictive model to the validation set. The Hosmer-Lemeshow test demonstrated a high level of agreement between the predicted probabilities and the actual probabilities (training set, P = 0.291; validation set, P = 0.284) (Fig. 4). DCA indicated that the threshold probabilities for the predictive model were between 10 and 92% for the training data and 12–82% for the validation data (Fig. 5).

## Discussion

Lymphedema, a recurrent and often intractable swelling disorder, severely impacts the quality of life for postoperative breast cancer patients and presents substantial barriers to effective treatment^21^. The incidence of BCRL peaks at 12 to 30 months post-surgery^8^, with about 90% of lymphedema cases occurring within 24 months after surgery^22^. Timely intervention in addressing risk contributors may lead to a 50% decline in the rate of onset^23^.

Our study successfully constructed and validated a predictive model for assessing the risk of BCRL. By analyzing data from 1485 patients, we identified operative time, BMI, the number of lymph nodes dissected, level of ALND, surgical site infection, and radiotherapy as independent risk factors for BCRL. Our research is the first, as far as we know, to establish operative time as a key independent variable in constructing and validating a nomogram to predict BCRL.

This study introduces a novel perspective on the significance of operative time as a critical risk factor for BCRL, which has not been previously emphasized in the literature. The identification of operative time as a substantial independent variable provides a new dimension in understanding the multifactorial nature of BCRL development. This finding suggests that surgical techniques and durations could be targeted for improvement to mitigate the risk of lymphedema, offering a practical intervention point for surgeons. Furthermore, integrating operative time with established factors like BMI, number of lymph nodes dissected, level of ALND, surgical site infection, and radiotherapy, we provide a more comprehensive predictive model. This integrative approach underscores the multifactorial etiology of BCRL and highlights the need for a multidisciplinary strategy in prevention and management.

Operative time was found to be a significant risk factor for BCRL. Our study demonstrated that patients with operative times exceeding 2 h had a significantly higher risk of developing BCRL compared to those with operative times under 2 h (OR 2.85; 95% CI 1.98–4.12). Prolonged operative time may lead to greater tissue damage and inflammatory response during surgery^24^, which can compromise the function of the lymphatic system and result in obstructed lymph flow^25,26^. Extended operative time might be due to increased surgical complexity, which could relate to broader surgical scope, heightened technical difficulty, or patient-specific anatomical considerations^27,28^. Therefore, reducing operative time in clinical practice may help lower the risk of BCRL, which requires physicians to consider time-saving measures during surgical planning and execution without compromising the safety and thoroughness of tumor removal.

BMI, the number of lymph nodes dissected, level of ALND, surgical site infection, and radiotherapy are five predictive factors commonly found in other risk prediction models^7,8,29,30^. Elevated BMI stands as a solitary predictor of BCRL in the postoperative period. Considering the diversity in populations studied and the diagnostic standards applied, the accepted BMI reference range is established between 25 and 30 kg/m^2^ ref^31^. Although there is controversy over the BMI standard range, maintaining weight within recommended limits is essential for patient care, necessitating healthcare professionals to facilitate patient education on post-surgical weight management as a strategy to decrease the incidence of lymphedema^32^. Studies have substantiated that extensive lymph node dissection and the excision of a larger quantity of lymph nodes correlate with a heightened incidence of lymphedema^16,33^, due to more disruption or reduction of lymphatic reflux pathways. Our study showed that a lymph node count > 20 (OR 5.65; 95% CI 3.50–9.13) and level III of ALND (OR 7.89; 95% CI 2.81–16.33) were the two strongest predictive factors for lymphedema, highlighting the imperative for healthcare professionals to acknowledge the significance of lymphatic tissue conservation and strive for maximal preservation during operative procedures to reduce the potential for lymphedema onset. The presence of surgical site infection significantly increases the risk of BCRL^33^. Infections may cause local inflammatory responses and scarring, affecting lymphatic return and leading to lymphedema. Therefore, postoperative anti-infection measures and proper incision care are crucial for BCRL prevention. Our research further revealed that radiotherapy stands as an independent predictor for the development of BCRL, with an odds ratio of 2.28 (95% CI 1.72–3.03). Johnson et al. found that radiotherapy to the regional lymph nodes following ALND may lead to a 19.3% increased incidence of BCRL^34^. Extensive radiotherapy is associated with the expansion and fibrotic changes in lymphatic channels. As breast-conserving surgery becomes more widespread, the risk associated with radiotherapy also increases^35^.

This study established a predictive model based on retrospective cohort study data. Across the training and validation cohorts, the model demonstrated good discriminative ability, evidenced by ROC curve areas of 0.779 and 0.724, respectively. This suggests that the model possesses a moderate degree of precision in stratifying patients by high and low risk. Additionally, the model’s calibration was well substantiated by the calibration curve analysis, and consistency between anticipated and actual event probabilities was confirmed by the Hosmer-Lemeshow test outcomes. This suggests that the model can not only accurately predict the risk of BCRL but also has generalizability across different patient populations. DCA provided us with important insights into the clinical application value of the model. DCA indicated that clinical decisions made using the model within its threshold probability range could yield net benefits.

Our model is built upon a selection of clinical and therapeutic factors that are essential for the effective surveillance and early intervention of lymphedema, aiming to decrease its prevalence. Efforts to manage BCRL are geared towards early detection and preventive care. Lymphedema is categorized into four stages by the International Society of Lymphology. Stages 0 and I are the earliest and can be reversed, involving fluid buildup without tissue hardening. Stages II and III are advanced and irreversible, with tissue becoming fibrous^36,37^. BCRL has no complete cure, treatments can be costly, and early detection is crucial for better outcomes. Without prompt treatment, swelling and tissue damage may become persistent^38^. Hence, early detection of lymphedema risk factors is essential for its prevention and early surveillance.

However, there are some limitations to our study. First, this is a retrospective single-center study, which may be subject to selection bias and information bias. Second, some factors that may influence the risk of BCRL, such as genetic predisposition, patient lifestyle, and precise radiation dose, were not included in the model. Future studies should consider these variables to further optimize prediction models. Additionally, there is a potential issue of multicollinearity among the variables such as the number of lymph nodes removed, ALND level, and operative time, which could affect the robustness of our findings. To address this, future research could employ advanced statistical techniques like structural equation modeling (SEM) or partial least squares regression (PLS) to better manage multicollinearity. Moreover, the use of a 2 cm circumference difference as a diagnostic criterion for lymphoedema may lead to both false positive and false negative diagnoses, given the lack of a universally accepted gold standard. This limitation underscores the need for more precise and evidence-based diagnostic criteria. Finally, our prediction model was validated at a single medical center, and its applicability to other medical centers and different patient populations requires further investigation.

## Conclusion

This study identified operative time, BMI, the number of lymph nodes dissected, level of ALND, surgical site infection, and radiotherapy as independent risk factors for BCRL, with the number of lymph nodes dissected and the level of ALND being the two strongest predictive factors. Moreover, for the first time, we combined operative time with BMI, the number of lymph nodes dissected, level of ALND, surgical site infection, and radiotherapy to design a nomogram that can conveniently assess the risk of BCRL. Nevertheless, further studies in larger, multicentric, and prospective cohorts are needed to validate these findings.

### Supplementary Information

Supplementary Table 1.

## Data Availability

The primary data can be acquired from the corresponding authors in compliance with privacy and ethical constraints.
