# Supplementary material for: Development and validation of a nomogram for breast cancer-related lymphedema
Source: Sci Rep. 2024 Jul 6;14:15602. doi: 10.1038/s41598-024-66573-1 (PMC11227568; doi:10.1038/s41598-024-66573-1)
Supplement: Supplementary file 1 — Supplementary Table 1. [file 41598_2024_66573_MOESM1_ESM.docx]

Supplementary Table 1. Detailed Treatment Characteristics and Patient Demographics for the Study Participants.

| Characteristic | Total  (n = 1485), N (%) | Training set  (n = 1038), N (%) | Validation set  (n = 447), N (%) | Lymphoedema  (n = 360), N (%) | Non-lymphoedema  (n = 1125), N (%) | P  Le vs. non-le |
| --- | --- | --- | --- | --- | --- | --- |
| Type of Chemotherapy  Anthracyclines  Taxanes  Cyclophosphamide  Timing of Chemotherapy  Neoadjuvant  Adjuvant  Both | 600 (40.4)  500 (33.7)  310 (20.9)  700 (47.1)  710 (47.8)  70 (4.7) | 420 (40.5)  341 (32.8)  211 (20.3)  490 (47.2)  494 (47.6)  48 (4.6) | 180 (40.3)  149 (33.3)  89 (19.9)  210 (47.0)  216 (48.3)  22 (4.9) | 155 (43.1)  125 (34.7)  65 (18.1)  160 (44.4)  180 (50.0)  5 (1.4) | 445 (39.6)  375 (33.3)  245 (21.8)  551 (49.0)  550 (48.9)  24 (2.1) | 0.825  0.851  0.752  0.134  0.714  0.374 |
| Area of Radiotherapy  Breast only  Axillary only  Breast and Axillary | 245 (16.5)  74 (5.0)  74 (5.0) | 163 (15.7)  52 (5.0)  46 (4.4) | 82 (18.3)  22 (4.9)  28 (6.3) | 60 (16.7)  35 (9.7)  52 (14.4) | 185 (16.4)  39 (3.5)  22 (2.0) | 0.921  < 0.001  < 0.001 |

Le Lymphoedema, non-le non-lymphoedema.
